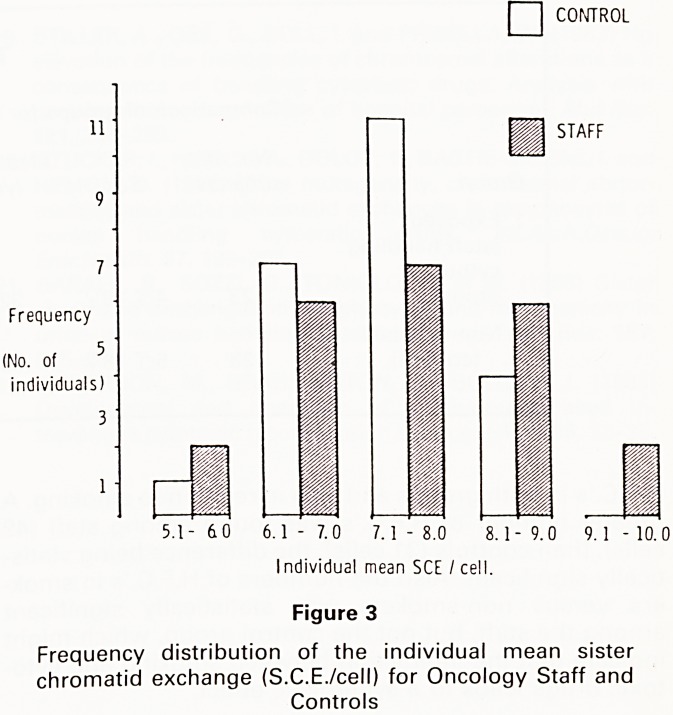# Staff Hazards in Handling Chemotherapy

**Published:** 1988

**Authors:** S. Ritha, R. Howell, M. G. Mott

**Affiliations:** Bristol Children's Hospital; Bristol Children's Hospital; Bristol Children's Hospital


### Hazards to Paediatric Oncology Staff Handling Chemotherapy

**S. Ritha; R. Howell, M. G. Mott** Bristol Children's Hospital

### INTRODUCTION

In recent years, there has been a huge increase in the use of antineoplastic agents. The past 20 years has seen fundamental advances in the chemotherapy of neoplastic diseases: the number of effective cytotoxic drugs has increased (30 cancer chemotherapeutic agents are commercially available, and another 70 are in some stage of clinical development), but the greatest progress has been in the design of more effective regimens for concurrent administration of drugs. By 1965, significant palliative results had been achieved with chemotherapy, but today we can list a substantial number of neoplastic diseases that can be cured. New strategies in antineoplastic therapy have been applied during the last two decades to children with malignant diseases, which often consist of treatment with as many effective agents as possible in the highest doses possible. Also many cancers of children require chemotherapy for a period of many years before cure can be achieved. This increased use has led to short term and long term consequences of chemotherapy that are now attracting much interest.

Unfortunately, many chemotherapeutic agents are known or suspected to be mutagenic/carcinogenic in animals and in patients treated with therapeutic doses (1,2). The International Agency for Research on Cancer of the W.H.O. has identifed at least five cancer chemotherapeutic agents with definite evidence of carcinogenicity in animals (Adrlamycin, B.C.N.U., C.C.N.U., Cyclophosphamide and Dacarbazine). Broadly speaking drugs that act by alkylation and/or binding tightly to D.N.A. seem more likely to be carcinogens.

There are numerous case reports of cancer arising in patients treated with cancer therapy (3–6) which point to an increased risk of second cancer in survivors who have been cured of their first malignancy. Certain drugs in particular have been implicated in the development of secondary tumour in patients treated by chemotherapy (3, 4). The mutagenicity of these drugs has long been regarded as of little risk to any but the patients. However, increasing exposure and handling of these drugs by staff has led to growing concern over the past few years about the long term potential hazard of low level exposure to cytotoxic drugs (7, 8). The possibility of health hazard from handling antineoplastic drugs was first raised in 1970 (7). The risk to personnel involved in reconstituting, preparing and administering cytotoxic drugs can be summarised as:

1. local effects caused by direct contact with the skin, eyes and mucous membranes.
2. systemic, short or long-term effects, mainly due to inhalation, when an aerosol or airborne dust is generated during preparation. e.g. reports of nausea and headache, or liver damage, described in nurses working on an oncology ward (9).

Cytogenetic methods are used increasingly for monitoring exposure to potential mutagens/carcinogens in the environment (10, 11). The sister chromatid exchange (S.C.E.) assay is widely accepted as a particularly sensitive method for assessing chromosome damage induced by chemical agents (12, 13) since altered S.C.E. frequency can be recorded at mutagen levels too low to produce classical gross chromosomal abnormalities.

### SISTER CHROMATID EXCHANGE (S.C.E.)

S.C.E. has been defined as a symmetrical exchange of chromatin (genetic material) between the two sister chromatids that make up a chromosome, which does not result in an alteration in the overall chromosome morphology. However, S.C.E.'s can be visualised by a special staining technique, which involves the incorporation of a thymidine analogue, 5-bromodeoxyuridine (Budr) into the cell, followed by staining with a flurochrome and Giemsa technique (F.P.G.) (14). This procedure results in differential staining (one light, one dark) of the two chromatids of a chromosome and it is then readily apparent when exchanges have occurred between them and they can be easily counted and scored (Fig 1).

Normally human lymphocytes show 5–10 S.C.E./cell. Although the exact mechanism of S.C.E. formation is unknown, it may be considered as a reflection of damage and subsequent repair processes that occurred in the D.N.A. It has also been suggested that the S.C.F. is a means by which the cell copes with D.N.A. damage, so increased damage leads to increased S.C.F.

**Figure 1**

Differential staining of a Metaphase spread, showing sister chromatid exchanges

### THE STUDY

In order to evaluate the possible occupational hazards of handling cytotoxic agents in the Paediatric Oncology unit, S.C.E.'s were studied in peripheral blood lymphocytes of nurses and doctors. The study was performed on 23 nursing and medical staff, currently working in the paediatric oncology unit, who had been regularly handling anti-neoplastic drugs and were involved in intravenous treatment of patients.

The use of protective measures was not uniform among the group. Some used only gloves, others gloves, goggles, gown, plastic apron and mask. The controls were 23 healthy donors from the Blood Transfusion Centre, matched for age and sex, none of whom had been exposed to cytotoxic drugs. Information abut the state of health, medication, smoking habits, previous diagnostic X-ray or therapeutic irradiation, was collected on all subjects, as the S.C.E. frequency may be influenced by these external factors. There were no age/sex differences between the 2 groups and the number of smokers was equal. The number reporting diagnostic X-rays was comparable and none had received therapeutic irradiation. There were two on contraceptive pills in the exposed group, one on insulin, one on phenytoin, one on a diuretic and one on atenolol. Three controls were on contraceptive pills (Table I).

For S.C.E. analysic, 4 ml of venous blood was collected from each person in a heparinised bottle, equal number of samples for staff and control being processed on the same day to ensure identical culture conditions. Within a few hours of collection, whole blood cultures were established according to standard techniques: these involve using phytohaemagglutinin to stimulate the lymphocytes to divide in the presence of (Budr); as they pass through mitosis, they are trapped in metaphase by treatment with colchicine. Cells were harvested using a standard protocol, air dried slides were prepared and aged for at least one week before staining, as freshly made slides were found to show G-banding, rather than harlequin staining.

**Figure 2**

Distribution of individual mean S.C.E. in Oncology Staff and Controls. Means of the two groups are indicated by arrows

**Figure 3**

Frequency distribution of the individual mean sister chromatid exchange (S.C.E./cell) for Oncology Staff and Controls

The analysis of sister chromatid exchanges (S.C.E.) was carried out blind on coded slides by one person. One sister chromatid exchange was counted each time two adjacent segments of one of the chromatid in a chromosome were stained differentially (Fig 1). A minimum of 30 second division metaphases from each individual were analysed, the total number of cells analysed being 779 cells in the exposed group, and 803 cells in the controls. Two parameters were considered in analysing the data. The mean S.C.E./cell was calculated for each of the 46 individuals, and the mean S.C.E./cell for each person and for the two groups is illustrated in Fig. II. The mean S.C.E. was 7.59 (S.D.±3.3) among the oncology staff and 7.25 (S.D.±3.0) among the controls, the difference being not statistically significant.

However, of the twelve individuals with S.C.E. frequency higher than 8 per cell, eight were staff (35%) and only four were controls (17%), (Figure 3), revealing a tendency towards higher individual S.C.E. rate among the staff as compared to the controls. The other parameter was to consider individual cells in both groups, S.C.E. frequency in individual cells did not follow a normal distribution but was positively skewed owing to a small number of cells with a particularly high S.C.E. rate. The appearance of a subpopulation of lymphocytes with increased S.C.E. (referred to as "high frequency cells" or H.F.C.'s), was noticed in earlier studies (15, 16). These H.F.C's were defined as those with a count above 13, this figure being obtained from the control group, in whom the mean S.C.E./cell was 7.25, the pooled SD was 3.04 and 2 SD above the mean was 13. Table II shows the number of

**Table 1**

**Comparison of groups for factors which may influence SCE**

| *Group* | *No. of subjects* | *Sex* | *Mean age (yrs)* | *No. of Smokers* | *No. Reporting Diagnostic X-ray* | *No. Reporting Drug intake* |
|---|---|---|---|---|---|---|
| 1. Exposed (staff handling cytotoxic drugs) | 23 | 4♂ 19♀ | 32.9 | 4 (5–20/day) | 4 | 6 |
| 2. Non-exposed (control) | 23 | 5♂ 18♀ | 33.8 | 4 (4–15/day) | 2 | 3 |

H.F.C.'s in both groups and also in relation to smoking. A greater number of H.F.C.'s was found among staff (49 cells), than controls (31 cells), the difference being statistically significant. Also the numbers of H.F.C.'s in smokers versus non-smokers was statistically significant among the staff, but not the control group, which might indicate that the interaction between smoking and cytotoxic drugs leads to a synergistic effect.

### DISCUSSION

Although it is accepted that S.C.E. is a sensitive measure for detecting mutagenic exposure, any increase in S.C.E. associated with occupational exposure is likely to be small and difficult to detect. Two previous studies among nurses handling cytotoxic drugs showed an increased frequency of S.C.E. which was attributed to occupational exposure (17, 18), while similar studies showed no increase in S.C.E. (19–21). In our study the mean S.C.E./cell for staff was not significantly different from the mean S.C.E./cell for controls, which is in agreement with some of the previous studies. However, the study showed an excess number of staff with a mean S.C.E. frequency above 8, and also a significant increase in the number of H.F.C.'s among staff handling cytotoxic drugs when compared to controls, observations which may indicate a possible health hazard, and which emphasize the need for the most careful handling of cytotoxic drugs. A pharmacy based intravenous cytotoxic reconstitution service in specialist oncology unit (22) is increasingly recognised to be a necessary safeguard for the health of professional staff, and such a unit has been opened by us since this study was undertaken.

**Table II**

**Distribution of cells with high sister chromatid exchange levels (HFCs).**

| *Individuals* | *No. of cells with high SCE (>13)* | *All other cells* | *Total No. of cells* |
|---|---|---|---|
| *Exposed* | 49 | 730 | 779 |
| Smoker | 18 | 129 | |
| Non-smoker | 31 | 601 | |
| *Non-Exposed* | 31 | 772 | 803 |
| Smoker | 7 | 142 | |
| Non-smoker | 24 | 630 | |
| Total | 80 | 1502 | 1582 |

In the pharmacy the preparation of individualised doses of cytotoxic drugs for each patient within laminar-flow hoods and the disposal of unused drugs, reduces any risks to the minimum. In non-specialist hospitals, where exposure tends to be intermittent and of short duration, the risk to personnel will not be great, provided adequate precautions are followed. There are at present no specific legal requirements on the handling of cytotoxic drugs in this country. The only country that has issued government regulations to date is Norway.

Exposure to the excreta of patients undergoing anti-cancer treatment may represent an additional hazard, and monitoring of parental exposure to childhood patients needs to be considered.

Finally, it is recognised that the analysis of sister chromatid exchanges as an indicator of D.N.A. damage resulting from exposure to low concentration of antineoplastic agents has its limitations. We believe therefore that there is a need to look for more sensitive methods for evaluation and monitoring such exposure.

### REFERENCES

1. IARC. (1982) IARC Monographs on the evaluation of carcinogenic risk of chemicals to humans. *Supplement n.* **4**, Lyon, France.
2. HARRIS, C. E. (1976) The carcinogenicity of anti-cancer drugs: A hazard in man. *Cancer* **37**, 1014–1023.
3. CASCIATO, D. A. and SCOTT, J. L. (1979) Acute Leukemia following prolonged cytotoxic agent therapy—Medicine **58**, 32–47.
4. VALAGUSSA, P., SANTORO, A., KENDA, R. *et al.* (1980) Second malignancies in Hogkin's disease: accompolication of certain forms of treatment. *Brit.Med.J.* **280**, 216–219.
5. KRIKORIAN, J. G., BURKE, J. S., ROSENBERG, S. A. and KAPLAN, H. S. (1979) Occurence of non-Hodgkin's lymphoma after therapy for Hodgkin's disease. *New.Engl.J.Med.* **300**, 452–458.
6. Second malignancy in lymphoma patients. (1985) *The Lancet* **ii**, 1163–1164.
7. LAWRENCE, M. N. G. (1970) Possible hazards of handling anti-neoplastic drugs. *Paediatrics* **46**, 648–649.
8. KNOWLES, R. S., VIRDEN, J. E. (1980) Handling of injectable anti-neoplastic agent. *Brit.Med.J.* **281**, 589–591.
9. ASOTANIEMI, E. A., SUTINEN, S., ARRANTO, A. J. *et al.* (1983) Liver damage in nurses handling cytostatic agents. *Acta.Med.Scand.* **214**, 181–189.
10. LIANG, J. C. (1983) Cytogenetics and public health—Assays for environmental mutagens. The Cancer Bulletin; **35**, 138–143.
11. CHERRY, L. M. (1983) Cytogenetics and public health—Human mutagen exposure and the risk of disease. *The Cancer Bulletin* **35**, 144–149.
12. PERRY, P., EVANS, H. J. (1975) Cytological detection of mutagen/carcinogen exposure by sister chromatid exchange. *Nature* **258**, 121–125.

13. RAPOSA, T. (1978) Sister Chromatid Exchange studies for monitoring DNA damage and repair capacity after cytotoxic in vitro and in lymphocyte of leukemic patients under cytotoxic therapy. *Mutat.Res.* **57**, 241–251.
14. PERRY, P. and WOLFF, S. (1974) New Giemsa method for the differential staining of sister chromatids. *Nature* **251**, 156–158.
15. STEKTA, D.G., MINKLER, J. and CARRANO, A. V. (1978) Induction of long-lived chromosome damage as manifested by sister chromatid exchange, in lymphocytes of animals exposed to mitomycin C. *Mut.Res.* **51**, 383–396.
16. CARRANO, A. V., MOORE, D. H. (1982) The rationale and methodology for quantifying SCE in humans. Mutagenicity: *New Horizons in genetic toxicity,* London: Academic Press.
17. NORPPA, H., SORSA, M., VIANIO, H. *et al.* (1980) Increased sister chromatid exchange frequencies in lymphocytes of nurses handling cytostatic drugs. *Scand.J.Work.Environ.Health.* **6**, 299–301.
18. WAKSVIK, H., KLEPP, O. and BOGGER, A. (1981) Chromosome analysis of nurses handling cytostatic agents. *Cancer treat. Rep.* **65**, 607–610.
19. STILLER, A., OBE, G., BOLL, I. and PRIBELLA, W. (1983) No elevation of the frequencies of chromosmal alterations as a consequence of handling cytostatic drugs. Analysis with peripheral blood and urine of hospital personnel. *Mut.Res.* **121**, 253–259.
20. STUCKER, I., HIRSCH, A., DOLOY, T., BASTIE-SIGEAC, I. and HEMON, D. (1986) Urine mutagencity, chromosmal abnormalities and sister chromatid exchanges in lymphocytes of nurses handling cytostatic drugs. *Int.Arch.Occup. Enir.Health.* **57**, 195–205.
21. BARALE, R., SOZZI, G., TONIOLO, P. *et al.* (1985) Sister chromatid exchanges in lymphocytes and mutagenicity in urine of nurses handling cytostatic drugs. *Mur.Res.* **157**, 235–240.
22. ANDERSON, M., BRASSINGTON, D., BOLGER, J. (1983) Development and operation of a pharmacy-based intravenous cytotoxic reconstitution service. *BMJ* **286**, 32–35.

## Figures and Tables

**Figure 1 f1:**
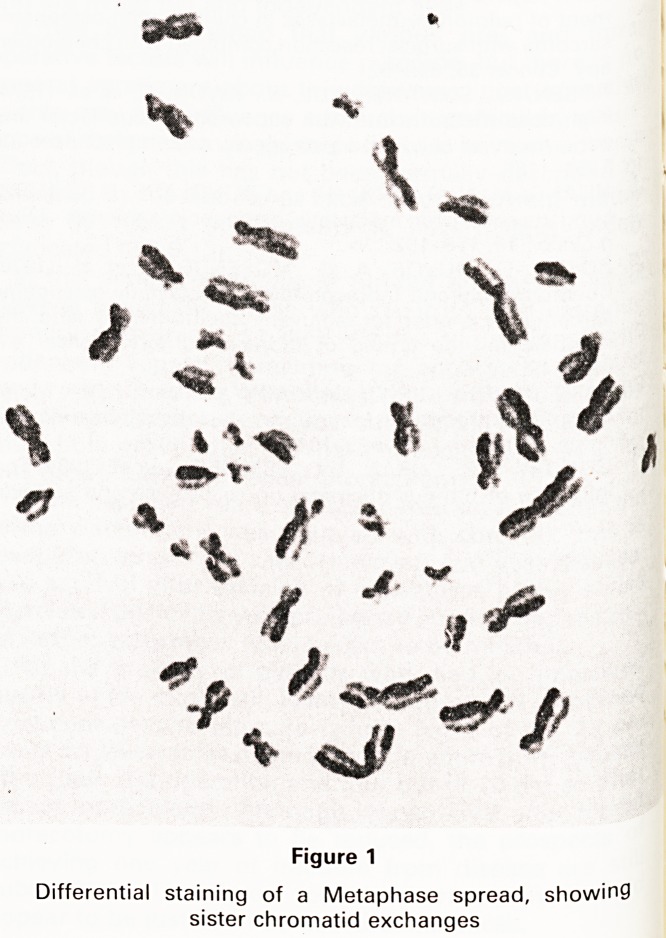


**Figure 2 f2:**
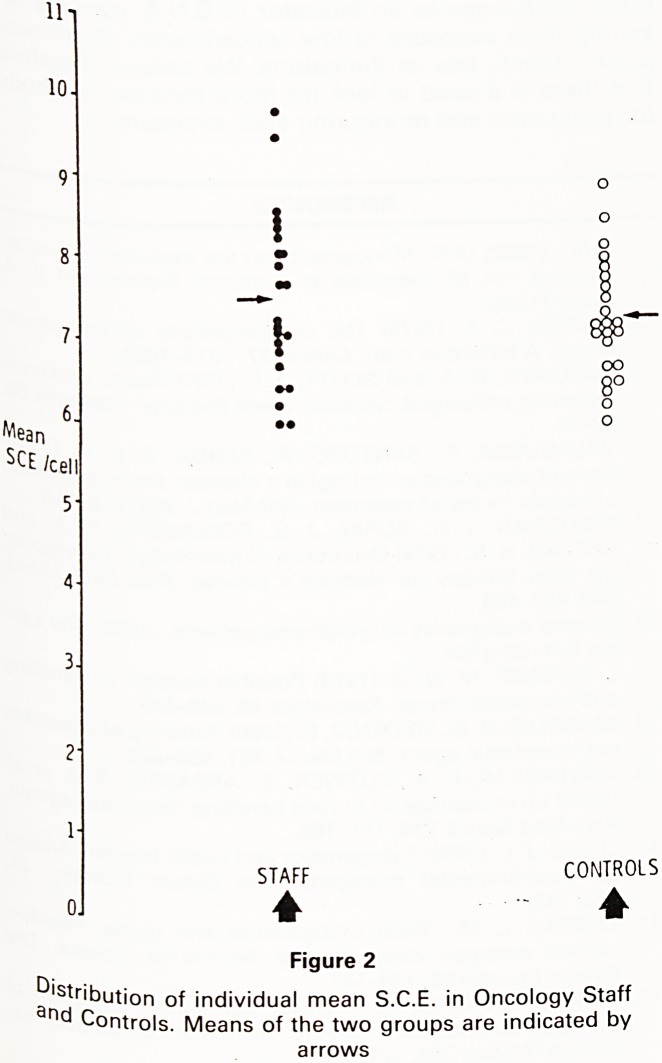


**Figure 3 f3:**